# Effect of vaginal pH on the efficacy of the dinoprostone vaginal insert for cervical ripening in patients with an unfavorable bishop score

**DOI:** 10.61622/rbgo/2025rbgo96

**Published:** 2025-12-09

**Authors:** Sílvia Serrano, Inês Martins, Marina Gato, Carina Simões, Andreia Fonseca, Helena Ferreira, Mónica Centeno

**Affiliations:** 1 Centro Hospitalar Universitário Lisboa Norte Hospital de Santa Maria Lisbon Portugal Centro Hospitalar Universitário Lisboa Norte, Hospital de Santa Maria, Lisbon, Portugal.; 2 Lisbon School of Medicine Lisbon Academic Medical Center Lisbon Portugal Lisbon School of Medicine, Lisbon Academic Medical Center, Lisbon, Portugal.

**Keywords:** Dinoprostone, Vaginal pH, Bishop's Score, Cervical ripening

## Abstract

**Objective:**

The aim of this study was to evaluate the effect of vaginal pH on the efficacy of the dinoprostone vaginal insert for cervical ripening in patients with unfavorable Bishop Score.

**Methods:**

Prospective observational cohort study, conducted at a Portuguese tertiary hospital. Term pregnant women with a singleton pregnancy and vertex presentation who had indication for labor induction with Bishop's Score ≤ 6 were included. Vaginal pH was measured with high accuracy strips before vaginal examination for Bishop Score was performed. The intravaginal dinoprostone insert (Propess^®^) was then applied into the posterior fornix. The primary outcome was the change of the Bishop's Score before placement and after removal of the dinoprostone insert. Spearman correlation was analyzed (SPSS 16.0).

**Results:**

A total of 75 women were included, with a gestational age of 39 ± 1,2 weeks. Mean initial vaginal pH was 4.67 ± 0.51. Mean variation of Bishop Score was 1.8 ± 2.3 points. There was a weak correlation between vaginal pH and changes on the Bishop Score with dinoprostone vaginal insert use (ρ = −0.123; p = 0.3).

**Conclusion:**

Vaginal pH does not appear to be a major influencer of the efficacy of dinoprostone vaginal insert for cervical ripening.

## Introduction

Labor induction can be required in up to 30% of pregnancies.^(1)^ Cervical ripening aims to shorten the duration of labor induction and increase the possibility of vaginal delivery. If the Bishop Score is unfavorable, the cervical ripening is usually performed prior to induction to attempt a modification of the internal structure of the cervix to facilitate its effacement and dilation.

Numerous studies have demonstrated the effectiveness of prostaglandins in promoting cervical ripening and to facilitate labor induction.^(2,3)^ Most prostaglandins are short-lived and of transient existence when produced endogenously, but some synthetic analogues of naturally occurring isoforms, such as prostaglandin (PG) E1, E2 and F2a, are stable enough to enable therapeutic utility.^(4)^ The most widely used synthetic version of prostaglandin E2 is known as dinoprostone.^(5)^ Dinoprostone is a synthetic analog of PG E2 (PGE2) that has been used in since the 1960s.^(3)^ It is commercially available in several forms including tablets, gels, suppositories, and pessaries.^(6)^ Prostin E2 tablets contain 3 mg of dinoprostone have a similar rate of delivery within 24h and mode of delivery compared with Prostin gel based on a previous study.^(7)^ Prostin E2 gel (Prepidil^®^) was the most commonly used form in the United Kingdom with a 3–5% failure rate, although it may precipitate uterine hyperstimulation and a nonreassuring fetal heart rate.^(8)^ Thus, the alternative for Prostin gel, it is the dinoprostone vaginal insert (Propess^®^), that were sought because of this situation.^(9)^

The dinoprostone vaginal insert delivers a single 10 mg dose of dinoprostone via a controlled-release system at 0.3 mg/h.^(10)^ Several studies have demonstrated that dinoprostone vaginal insert can effectively ripen the cervix and promote uterine contractions.^(4)^ The use of this method has the advantage that it can be easily removed in cases of uterine tachysystole and suspicious or pathological cardiotocographic changes. A vaginal pessary is considered easier to administer, less invasive, and cost-effective compared with an instant-release pessary.^(9)^

Although clinically effective, wide variability in observed efficacy between patients is occasionally noted. The factors that explain these variations are not clarified. Recent studies have suggested that vaginal pH may account for the variability on clinical efficacy.^(11-14)^ The normal vaginal pH varies between 3.8-4.5 but a variety of factors can modify it.^(15)^ Whether the vaginal pH may alter the release of the dinoprostone is unclear.

The aim of this study was to evaluate the effect of vaginal pH on the efficacy of dinoprostone vaginal insert for cervical ripening in patients with unfavorable Bishop Score.

## Methods

A prospective observational cohort study was performed from January to July 2021 at a tertiary care hospital in Lisbon, Portugal.

Based on the Law of Large Numbers, a sample size of at least 60 cases was determined to ensure statistical reliability and representativeness.

Pregnant women with a term singleton pregnancy, vertex presentation and intact membranes who had indication for labor induction with Bishop's Score ≤ 6 were included.

Indications for induction included reaching 41 weeks of gestation. Induction could also be done earlier if there were maternal conditions such as hypertensive disorders or diabetes. Fetal conditions like suspected macrosomia or fetal growth restriction also justified earlier induction.

Women with previous uterine surgery (such as a prior cesarean section or myomectomy involving entry into the uterine cavity), a suspected or pathological cardiotocographic registering, uterine tachysystole, drug hypersensitivity, glaucoma or any contraindication to vaginal birth were excluded.

Maternal and obstetrical characteristics such as age and BMI, maternal and fetal pathology, parity, number of vaginal deliveries and gestational age at delivery were recorded.

The local protocol for cervical ripening was followed. A cardiotocography (CTG) was performed for 20 min before the procedure, if the result was normal the procedure was continued. Vaginal pH was measured with color strips (Merck^®^). The pH indicator strip was inserted into the middle third of the vagina and pressed against the walls of the vagina for 60 seconds to ensure that the strip was well dripping with vaginal secretions. The result was obtained immediately; the change color of the strip was compared with the established scale. A vaginal examination was then performed by an obstetrician, obstetrical resident or designated midwife and the Bishop Score assessed and registered. Women with Bishop Score <6 received a dinoprostone vaginal insert 10 mg inserted into the posterior fornix. A follow up CTG was performed for one hour, and repeated 3 times per day. The insert was removed in the following circumstances: 24 hours after insertion; labor; uterine tachysystole; nonreassuring fetal heart rate monitoring; other adverse events (such as hemorrhage). Cervical reassessment and Bishop score reevaluation were repeated after exteriorization of the insert. All decisions regarding amniotomy, oxytocin augmentation and analgesia were decided by the attending physician.

The primary outcome was the change of the Bishop's score before placement and after removal of the dinoprostone insert (measured as "score after" minus "score before"). Other maternal outcomes included the time interval from placement of the dinoprostone system to vaginal delivery and the type of delivery. Umbilical artery cord pH, Apgar score at 1 and 5 minutes and admission to neonatal intensive care unit (NICU) were the neonatal/fetal outcomes considered.

The study was thoroughly explained to the pregnant women, and informed consent was obtained prior to the assessment of vaginal pH. Furthermore, the study was approved by the institutional ethics committee for research on human subjects (CRE 367/21).

Descriptive analysis was used to study the demographic data. Averages and standard deviations were calculated for continuous variables. Frequency and percentage were calculated for categorical variables. Spearman correlation was analyzed for the primary outcome.

All statistical analyses were performed using IBM SPPS Statistics version 16.0, and two-sided p values < 0.05 were considered statistically significant.

## Results

**Figure 1:**
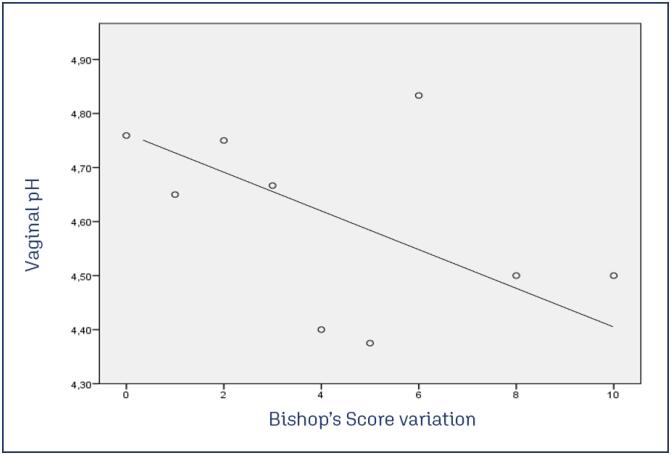
The scatter plot shows the relationship between vaginal pH and changes in Bishop's Score after using the dinoprostone insert. There was a weak negative correlation (Spearman's ρ = −0.123; p = 0.3), which is not statistically significant. Women with a vaginal pH of 4.5 or lower had bigger changes in Bishop's Score than women with pH above 4.5

Seventy-five women were included in the study. the mean gestational age was 39 ± 1.2 weeks and 40% were primigravida. Mean initial vaginal pH was 4.67 ± 0.51. A vaginal pH of ≤4.5 was observed in 47 participants (62%) Initial mean Bishop score was 3.0 ± 1.4 points. Baseline characteristics are described in table 1. Mean variation of Bishop Score was 1.8 ± 2.3 points, with a mean score of 4.8 ± 2.8 points after insert removal (Table 2). There was a weak negative correlation between vaginal pH and changes on the Bishop Score with dinoprostone insert (ρ= −0.123; p = 0.3) (Figure 1). The dinoprostone insert was in place for 14 ± 7,4h on average. The main reasons for removing the insert were spontaneous expulsion during ambulation, while using the bathroom, or following membrane rupture (29 cases, 39%), completion of 24 hours since insertion (19 cases, 25%), and the onset of labor (17 cases, 22%). Sixty women (80%) had a vaginal delivery, with the time from cervical ripening to vaginal delivery of 30±12h. Out of 75 deliveries, 5 babies had Apgar score < 7 at minute 5 of life but none needed to be admitted to the Neonatal Intensive Care Unit (NICU). There were no cases of neonatal mortality.

**Table 1:** Baseline characteristics

Characteristics	Mean ± SD or n(%)
Age (years)	32 ± 6.7
Gestagional age (weeks)	39 ± 1.2
Body mass index (Kg/m²)	27 ± 5.8
Parity	2.25 ± 1.1
Primiparous	30(40)
Multiparous	45(60)
Initial bishop score	3.0 ± 1.4
Vaginal pH	4.67 ± 0.51
pH between 3.5 and 4.5	47(62)
pH between 5 and 6	28(37)

**Table 2:** Effects of dinoprostone vaginal insert

Effects of vaginal dinoprostone	Mean ± SD
Bishop score after the insert removal	4.8 ± 2.8
Change in bishop score	1.8 ± 2.3
Application duration	14 ± 7.4h
**Reasons for removing the dinoprostone insert**	**n(%)**
24 hours after insertion	19(25)
Labor	17(22)
Tachysystole	2(3)
Nonreassuring fetal hear rate	6(8)
Spontaneous externalization of the insert	29(39)
Other non-specific adverse events	2(3)

**Table 3:** Delivery data after vaginal dinoprostone insert

Delivery outcomes		Mean ± SD or n(%)
Time to vaginal delivery		30 ± 12h
Vaginal delivery		60(80)
	Vacuum assisted delivery	17(23)
	Forceps	3(4)
	Vacuum + Forceps	2(3)
Cesarean delivery		15(20)

## Discussion

Our results suggest that vaginal pH does not appear to play a major role on the efficacy of dinoprostone vaginal insert for cervical ripening. Although the results were not significant, we found a weak negative correlation between vaginal pH and the Bishop's score change. In our study cohort, women with a lower vaginal pH (≤4.5) had greater variations of Bishop score compared to women with a higher pH (> 4.5).

Ramsey et al. in 2003 also assessed the effect of vaginal pH on the effectiveness of dinoprostone vaginal insert (CervidilÒ^®^) and bear out the fact that vaginal pH does not appear to influence the efficacy of the controlled-released dinoprostone vaginal insert for cervical ripening/labor induction.^(16)^

However, Lyrenas *et al* evaluated the effect of vaginal pH and efficacy of a controlled-release PGE2 vaginal insert in 68 subjects and noted the PGE2 release rate was dependent on vaginal pH, with a faster release rate at higher vaginal pH.^(11)^

Basirat Z et al. in their study found that the average active phase duration in patients with high pH was significantly shorter than those with low pH (p=0.019). In the study, the cesarean section rate, the Bishop score after 12 hours, latent phase and second stage durations were not statistically significant.^(12)^

Onen et al. in their study found that Bishop's score change over the initial 12 hours significantly differed between the low vaginal pH (3.9±3.3) and the high vaginal pH (5.5±3.4) group of women.^(13)^

There is no consensus among the results of the studies. Other surveys were developed to assess the relationship between vaginal pH and efficacy of some PGE2 preparations such as PGE2 gel. A study conducted to analyze the effect of vaginal pH on intracervical dinoprostone gel application showed that a higher vaginal pH was associated with a higher Bishop score prior to induction, and a higher number of vaginal deliveries than those with lower vaginal pH.^(14)^

Ramsey et al. studies conducted in 2002 and 2003 conflict each other.^(16,17)^ The study in 2002 conducted with PGE2 gel showed significant association between higher vaginal pH and the shorter time to active labor, complete dilation, and delivery while the study in 2003 conducted with PGE2 vaginal insert showed no significance.

Johnson et al. and Taylor et al. studied in vitro release of PGE2 from many commercially available preparations and reported an increased PGE2 release in solutions with a higher pH (6.5 to 7.5).^(18,19)^

The results of our study endorse those of a meta-analysis published in 2022, showing that the vaginal pH does not seem to affect the potency of vaginally administered prostaglandins. This meta-analysis showed that an acidic vaginal pH did not influence the efficacy of misoprostol or dinoprostone in terms of accomplishing a successful vaginal delivery (OR 0.62, 95% CI 0.29, 1.30) and that the interval to delivery was unaffected by the acidity of vaginal pH (Mean Difference 4.18 h, 95% CI −2.09, 10.45).^(15)^ However, different dinoprostone preparations may have different influences on the vaginal pH. One reason for this could be the use of different excipients in the controlled release dinoprostone delivery vehicle, which alters the way PGE2 is released.

In our study, the evaluation and follow-up according to an established protocol is a positive aspect that standardized clinical practice. Strict exclusion criteria were used, which helped to avoid confounding bias.

The present study is limited by the relatively small sample size and the small variation that was observed in the vaginal pH between patients. However, considering the low correlation coefficient between vaginal pH and Bishop score change, it is unlikely that an investigation with a larger sample size would demonstrate a significant result.

Although the sample size is small, this limitation does not undermine the validity of the study, as it is an exploratory investigation designed to gather preliminary data. The statistical techniques employed are appropriate for small sample sizes, ensuring meaningful and reliable results for future research. Presumably, a well-designed pharmacokinetic study with a larger sample size would be needed to thoroughly evaluate how changes in vaginal pH influence cervical ripening and labor induction, building on the insights provided by this preliminary investigation.

## Conclusion

In conclusion, vaginal pH does not appear to be a major influencer on the efficacy of dinoprostone vaginal insert (Propess^®^) for cervical ripening, evaluated by the change on the Bishop Score.

## Data availability

: The authors did not make the data from this article available in repositories prior to submission.

## References

[1] Yount SM, Lassiter N (2013). The pharmacology of prostaglandins for induction of labor. J Midwifery Womens Health.

[2] Hayashi RH (1993). Spontaneous and induced cervical ripening. Natural dilation and effacement process and current cervical ripening techniques. J Reprod Med.

[3] Keirse MJ (1993). Prostaglandins in preinduction cervical ripening. Meta-analysis of worldwide clinical experience. J Reprod Med.

[4] Pierce S, Bakker R, Myers DA, Edwards RK (2018). Clinical insights for cervical ripening and labor induction using prostaglandins. AJP Rep.

[5] Hawkins JS, Wing DA (2012). Current pharmacotherapy options for labor induction. Expert Opin Pharmacother.

[6] Thomas J, Fairclough A, Kavanagh J, Kelly AJ (2014). Vaginal prostaglandin (PGE2 and PGF2a) for induction of labour at term. Cochrane Database Syst Rev.

[7] Khan ZA, Abdul B, Majoko F (2011). Induction of labour with vaginal prostaglandin tablet vs gel. J Obstet Gynaecol.

[8] Vince K, Matijević R (2022). Comparison of intracervical and intravaginal prostaglandin E2 for induction of labor in term pregnancies with unfavorable cervix: randomized controlled trial. Eur J Obstet Gynecol Reprod Biol.

[9] Mukhopadhyay M, Lim KJ, Fairlie FM (2002). Is propess a better method of induction of labour in nulliparous women. J Obstet Gynaecol.

[10] Itoh H, Ishii K, Shigeta N, Itakura A, Hamada H, Nagamatsu T (2021). Efficacy and safety of controlled-release dinoprostone vaginal delivery system (PROPESS) in Japanese pregnant women requiring cervical ripening: results from a multicenter, randomized, double-blind, placebo-controlled phase III study. J Obstet Gynaecol Res.

[11] Lyrenäs S, Clason I, Ulmsten U (2001). In vivo controlled release of PGE2 from a vaginal insert (0.8 mm, 10 mg) during induction of labour. BJOG.

[12] Basirat Z, Barat SH, Ghanbarpour A, Golsorkhtabar-Amiri M (2012). Does vaginal pH affect the efficacy of dinoprostone in cervical ripening/labor duration?. Clin Exp Obstet Gynecol.

[13] Önen F, Özaksit G, Yilmaz B, Gungor T, Bilge Ü, Sut N (2008). The role of vaginal ph on efficacy of controlled-release dinoprostone vaginal insert for cervical ripening/labor induction: a prospective double-blind study. J Turk Ger Gynecol Assoc.

[14] Kurian M, Rao B, Rao AA (2016). Effect of vaginal pH on efficacy of dinoprostone gel for labour induction. Int J Reprod Contracept Obstet Gynecol.

[15] Pergialiotis V, Papadatou K, Panagiotopoulos M, Bellos I, Papapanagiotou A, Rodolakis A (2022). The impact of vaginal pH on induction of labour outcomes: a meta-analysis of observational studies. J Obstet Gynaecol.

[16] Ramsey PS, Ogburn PL, Harris DY, Heise RH, DiMarco CS, Ramin KD (2003). Effect of vaginal pH on efficacy of the controlled-release dinoprostone vaginal insert for cervical ripening/labor induction. J Matern Fetal Neonatal Med.

[17] Ramsey PS, Ogburn PL, Harris DY, Heise RH, Ramin KD (2002). Effect of vaginal pH on efficacy of the dinoprostone gel for cervical ripening/labor induction. Am J Obstet Gynecol.

[18] Johnson TA, Greer IA, Kelly RW, Calder AA (1992). The effect of pH on release of PGE2 from vaginal and endocervical preparations for induction of labour. Br J Obstet Gynaecol.

[19] Taylor AV, MacKenzie IZ (1993). The effect of pH on release of PGE2 from vaginal and endocervical preparations for induction of labour. BJOG.

